# Impact of Allergic Contact Dermatitis on the Quality of Life and Work Productivity

**DOI:** 10.1155/2019/3797536

**Published:** 2019-03-03

**Authors:** H. Kalboussi, I. Kacem, H. Aroui, O. El Maalel, M. Maoua, A. Brahem, S. El Guedri, S. Chatti, N. Ghariani, N. Mrizak

**Affiliations:** ^1^Occupational Medicine Department, Teaching Hospital Farhat Hached Sousse, Tunisia; ^2^Faculty of Medicine Ibn El Jazzar of Sousse, University of Sousse, Tunisia; ^3^Occupational Medicine Department, Teaching Hospital Ibn Jazzar Kairouan, Tunisia; ^4^Dermatology Department, Teaching Hospital Farhat Hached Sousse, Tunisia

## Abstract

**Background:**

Allergic contact dermatitis (ACD) is a common chronic skin disease that generates considerable public-health and socioeconomic costs. This disease affects the quality of life and the occupational activity of patients.

**Aims:**

To assess the quality of life (QOL) of patients with ACD and study the impact of this disease on their work productivity.

**Methods:**

This is a cross-sectional study carried out from January 2012 to December 2014. All patients diagnosed with ACD in the Dermato-Allergology Unit of the Occupational Medicine Department at Farhat Hached University Hospital, in Sousse, were included. The impact of skin disease on the QOL of affected persons was assessed using the Dermatology Life Quality Index (DLQI). The work productivity was measured using the Work Productivity and Activity Impairment Allergic Specific questionnaire (WPAI: AS).

**Results:**

The study population consisted of 150 patients. The average score of DLQI was 6.5. Over the previous 7 days, absenteeism rate was 25.9 ± 15.3%, presenteeism rate was 50.2 ± 32%, overall work productivity loss was 29.6 ± 19.4%, and daily activity impairment was 50.4 ± 32.3%. The DLQI score was significantly associated with atopy (p = 0.03), relapses strictly greater than 10 (p = 0.02), presenteeism (p <10^−3^), overall work productivity loss (p = 0.01), and daily activity impairment (p = 0.03).

**Conclusion:**

The impact of ACD on QOL and occupational activity seems important and requires specific attention from the occupational physician.

## 1. Introduction

Allergic contact dermatitis (ACD) is a common skin disease caused by a T-cell-mediated immune reaction to usually innocuous allergens [1]. It is an inflammatory reaction occurring at the site of challenge with a contact allergen in sensitized individuals. It is characterized by redness, papules, and vesicles, followed by scaling and dry skin [2].

This disease is considered as one of the most common dermatologic diseases and the primary cause of occupational skin diseases. Recent studies found that ACD could be responsible for 50 to 60% of occupational contact dermatitis (OCD) and 20 to 30 % of all occupational diseases [3, 4].

In Tunisia, its prevalence was 4% of all occupational diseases, according to a national study covering all the cases of occupational ACD recognized in the private sector from 2002 to 2012 [5].

The impact of ACD is often underestimated as it is not a life-threatening condition. It has been also considered as a trivial events related to job. However, many disabilities have been reported such as pain, itch, and psychosocial consequences [6]. All these factors can negatively affect the quality of life (QOL) of affected subjects.

The quality of life in ACD patients can be considered as a relatively new approach during consultation as it allows assessment and management of its impairment.

Moreover, the physical and psychosocial effects of this disease can have an important impact on the patients' occupational activity leading to more frequent absenteeism and more prolonged sick leaves than healthy workers involving the need to change occupation.

However, relatively few studies have been published concerning the impact of the ACD on the quality of life and on the occupational activity of the affected subjects and none have been carried out in Tunisia.

Thus, we have conducted this study to assess the quality of life of patients with ACD and analyze the impact of this disease on the work productivity in the affected subjects.

## 2. Methods

This is an epidemiological, cross-sectional study carried out over a 3 year period (from January 2012 to December 2014). We included all patients diagnosed with ACD in Dermato-Allergology Unit of the Occupational Medicine Department at Farhat Hached Teaching Hospital, in Sousse (Tunisia). Retired patients, unemployed ones, and students were excluded from the study.

The diagnosis of ACD was clinically made and confirmed by a relevant patch test.

Data was collected using a medical questionnaire and a dermatological clinical examination.The medical questionnaire explored the sociodemographic characteristics (age, gender, educational level, and marital status), occupational data (sector of activity, occupational seniority, and occupation), and medical information (family history of eczema, personal medical history, personal history of atopy (personal history of diagnosed allergic asthma or rhinitis or atopic eczema), duration of eczema evolution, number of relapses per year, symptoms, and prescription of treatment).

The dermatological clinical examination focused on the clinical appearance of ACD, its localization, and the extent of lesions.

We defined keratotic and lichenified lesions as chronic eczema, erythematous, and scaling as subacute and vesicular or bullous as acute lesions.

To evaluate the QOL of patients with ACD, we used the Dermatology Life Quality Index (DLQI) questionnaire [7]. It is a ten-question tool which assesses the impact of skin disease on the quality of life of an affected person. It was validated in people aged 16 years and above and having chronic hand dermatitis. It had been translated into more than 80 languages including Arabic [8, 9].

Scores range from 0 to 30 and the highest score indicate the most severe eczema. They are categorized as follows: 0-1 = no effect on the patient's life, 2-5 = low effect on the patient's life, 6-10 = moderate effect on the patient's life, 11-20 = significant effect on the patient's life, and 21-30 = extremely important effect on the patient's life [7, 10].

In order to assess productivity at work, we used the French version of the Work Productivity and Activity Impairment: Allergy Specific (WPAI:AS) Questionnaire. The WPAI questionnaire had been validated in several pathologies including chronic hand dermatitis [7, 9, 11].

It reflects the adaptation of employees to the demands of work in general, but also to the psychosocial context of the occupational environment in which they operate. It illustrates the possible dynamic imbalance between the employee and the work environment [9, 11].

The ‘Work Productivity and Activity Impairment Questionnaire' (WPAI) includes six questions to assess absenteeism, presenteeism, overall work productivity loss, and daily activity impairment due to ACD during the last seven days preceding the interview with the patient [12].

Results are multiplied by 100 and expressed as percentages of time lost. A higher percentage indicates greater depreciation and less productivity.

Absenteeism is defined as a temporary or permanent inability to work due to illness or infirmity [13]. Productivity is defined by the ratio between the quantities produced (or their added value, AV) and the means used to obtain those [14]. Presenteeism is characterized by the physical presence of the employee with productivity less than normally required.

Data were analyzed using SPSS 17.0 software. Categorical variables were expressed as numbers and percentages. Continuous variables were expressed as means and standard deviation, or median, and quartiles according to their distribution.

For the comparison of the means, we used the Student's t- test to compare two independent series averages and the Snedecor F-test of parametric variance analysis (one-way ANOVA) to compare several averages.

The comparison of frequencies was performed with the Pearson Chi square. The association between two quantitative variables was explored using the Pearson correlation coefficient. For multivariate analysis, we used a multiple binary logistic regression.

Independent variables were enrolled in the regression models when their degree of significance was less than 0.2. For all used tests, p value less than 0.05 was considered as statistically significant.

## 3. Results

During the study period, 150 patients with ACD were included. The average age was 38.9 ± 10.8 years. ACD was more frequent in men (55.3 %). The majority of patients were married (70%) and 59.4% had at least two children.

Twenty-three patients (15.3%) were working in construction field, twenty-one in the textile industry (14%), and nineteen (12.7%) in the healthcare sector. Average job seniority was 13.20 ± 9.16 years. Concerning the influence of ACD on work only 15 patients (10%) were transferred from their work station. Occupational data are summarized in Table 1.

Family history of eczema was found in 25 patients (16.7%). The presence of other skin diseases was found in 12 patients (8%).

The most common reported skin problems were vesicular lesions (19.3%) followed by scaling (9.3%). ACD extent was greater than or equal to 30% of body surface in thirty-one patients (20.7%).

ACD was localized in the hands and in the face respectively in 66% (n=99) and 7,7% (n=12) of cases. The average duration of eczema evolution was 24 months with extremes ranging between 8 and 75 months. Half of the patients (50%) had between one and five relapses per year. Medical data are summarized in Table 2.

**Figure 1:**
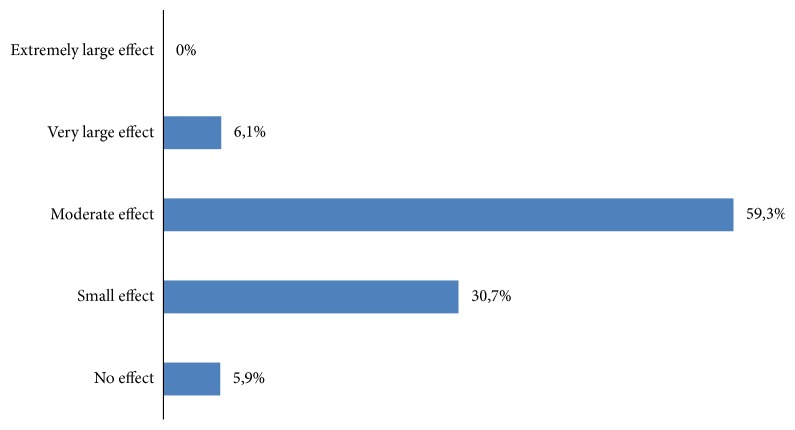
Classification of patients according to degree of the effect of eczema on their quality of life.

The average score of DLQI was 6.5 ± 2.7 with a range of 0 and 12. The majority of the patients reported a moderate effect of ACD on their lives (59.3%) (Figure 1).

Over the last seven days prior to completing the questionnaire, absenteeism rate was 25.9 ± 15.3%, presenteeism rate was 50.2 ± 32%, overall work productivity loss was 29.6 ± 19.4%, and daily activity impairment was 50.4 ± 32.3%.

The DLQI score was significantly more impaired in women than in men (7.02 versus 6.07) (*p*=0.035). It was also more impaired in patients with keratotic lesions and scaling (8) (*p* = 0.049). The DLQI score in patients with hand eczema was 6.36 and 5.95 in all other localizations with no significant association (*p*=0.14).

Absenteeism was significantly higher in patients aged above 50 years (*p* = 0.039) and an ACD extent above 19% of body surface (*p* = 0.037). It was significantly associated with DLQI score (*p*=0.001).

Presenteeism was associated with female gender (*p*=0.005), occupation (*p*=0.006), DLQI score (*p* <10^−3^), overall work productivity loss (*p* <10^−3^), and daily activity impairment (*p* <10^−3^).

Neither absenteeism nor presenteeism was significantly associated with hand eczema (*p*= 0,12 and* p*= 0,08, respectively).

Multivariate analysis showed that QOL was significantly associated with atopy (*p* = 0.03), relapses strictly greater than 10 (*p* = 0.02), presenteeism (*p* <10-3), overall work productivity loss (*p* = 0.01), and daily activity impairment (*p* = 0.03) (Table 3). Absenteeism was significantly associated with the extent of lesions> 30% of body surface area (*p* = 0.03), presenteeism (*p* <10^−3^), and overall work productivity loss (*p *<10^−3^) (Table 4).

Presenteeism was significantly associated with treatment (*p*=0.05) and the time interval between diagnosis and patch test with European standard baseline (*p* = 0.02). However, it was inversely proportional to the overall work productivity loss (*p* < 10^−3^) and the daily activity impairment (*p* <10^−3^) (Table 5).

## 4. Discussion

ACD is a common condition with an important socioeconomic cost because of its related personal and professional impairments.

This study was conducted to assess the impact of this dermatitis on the patients' QOL and occupational activity.

One hundred fifty patients meeting the inclusion criteria were enrolled. This acceptable sample can be improved by a multicenter approach or a longer study period. A control group can be needed as the cross-sectional study type could not allow establishing causal relationships.

The results of our study confirm the negative impact of ACD on patients' QOL as it has been already reported by Kadyk DL et al. [4], in a study using the modified Skindex-16 questionnaire.

It was difficult to compare our results with data in the literature. First, the majority of research regarding contact dermatitis includes patients with ICD as well. Second, the small amount of data available regarding outcomes in ACD was obtained using different dermatology specific QOL instruments. Each survey uses different questions and scoring to measure QOL.

In addition, the averages of DLQI scores of our patients were close to that found in some studies in patients with OCD [15–18].

When comparing ACD impact with that of other dermatological diseases, it was inferior to that of scars, burns, and epidermolysis bullosa [19, 20].

In our study, factors influencing QOL were gender, keratosic and erythemato-squamous lesions, atopy, and relapses strictly greater than 10.

In fact, several studies showed that the QOL of women is more impaired compared to that of men [21, 22]. These results could be explained by the characteristics of women, such as body image and psychological disturbances, as well as social acceptance [23]. However, Agner T et al. [16] did not find any significant difference between men and women concerning the quality of life, although men were more severely affected than women.

A history of atopy has been reported to be associated with poor QOL scores in patients with ACD [24, 25]. Controversial data are now available in the literature [4, 26].

Hand eczema was associated with impaired QOL [4]. A 5-year Australian cohort study, enrolling 119 people suffering from occupational ACD, revealed a significant correlation between the severity of the hand eczema and the QOL (p <10^−3^) as DLQI scores increased from 1 in the minimal forms to 5 in the moderate forms and to 11 in the severe forms [17].

Conversely, Thomsen SF et al. [27] found no association between the localization of contact dermatitis and QOL, which is consistent with our results.

Data related to the impact of dermatitis on QOL are limited and different tools were considered in the assessment of severity. Some authors considered objective criteria such as the appearance of lesions, their extent, and frequency of relapses [17, 18], while Matterne U et al. [28] used a simple description to determine severity such as the clinical appearance of the lesions.

Thomson KF et al. [29], in a study carried out in England, concluded to a significant relationship between delayed diagnosis and bad QOL (p = 0.004) and to an improvement of DLQI score by patch testing (p = 0.015). In fact, allergens identification allows its avoidance, hence the improvement of the QOL [4].

In the study of Kadyk DL et al. [4], assessing the QOL of 149 patients with ACD using Skindex-16, patients were particularly embarrassed by pruritus, irritation of the skin and the persistence of the allergy. When evaluating the 4 items of the considered questionnaire, the item of emotion had the worst score followed by symptoms (pruritus, burning, and pain), daily activities, and impact on work. In our study, the average DLQI score was high in patients who experienced itch with no statistically significant association (p = 0.17).

The relation between contact dermatitis and work involves the effect of professional practice on the disease and conversely, the impact of contact dermatitis on the professional activity.

Subjects who leave job due to skin disease had significantly more impaired QOL [30].

In our study, the results of WPAI: AS questionnaire have shown that over the last seven days before completing the questionnaire, absenteeism rate was 25.9 ± 15.3%, presenteeism rate was 50.2 ± 32%, overall work productivity loss was 29.6 ± 19.4%, and daily activity impairment was 50.4 ± 32.3%.

Previous studies have demonstrated the existence of a significant interruption of work in subjects with occupational ACD. Indeed, this pathology caused 10% of work stopping during the initial assessment and 26 to 38% of the judgments at 6 months of evaluation in the Holness DL study [31].

According to Clemmensen KKB et al. [32], in a cohort study performed in Denmark in 2010, about one-third of patients (n = 199) had lost their jobs, of whom 61% reported eczema as the cause.

In our study, the score of the QOL was significantly associated with presenteeism (p <10^−3^), an overall work productivity loss (p = 0.01) and an activity impairment (p = 0,03). Agner T et al. [16] found that QOL decreased when sick leave periods were prolonged (p<0.001).

Reilly TM et al. [9] showed that the average work time lost due to hand eczema was 0.3 ± 4% (n=196) presenteeism was 18 ± 22% (n = 197), overall work productivity loss was 17 ± 22% (n = 193), and activity impairment was 25 ± 25%. A multicentric European study found that 28% of patients with hand eczema were unable to work and 12% had an absence from work for more than 12 weeks [33].

In terms of presenteeism, absenteeism, and activity impairment, our results are higher than those of other investigations [11, 34–37]. The predominance of manual labour in our sample can explain this fact; it can be a predictor of sick leave. The relation between absenteeism, presenteeism, and disease also differs from one country to another because of the differences in social security systems [38].

## 5. Conclusion

The allergic contact dermatitis is still a hot topic as it is a frequent condition with a serious handicap because of its psychological, socioprofessional, and familial consequences.

It affects patients' quality of life and their occupational activity which must be considered by both occupational physicians and dermatologists.

Thus, a multidisciplinary approach combining a personalized education and a long-term follow-up are needed to improve the quality of life of patients with ACD.

## Figures and Tables

**Table 1:** Occupational characteristics of patients.

Variables	Number of cases	Percentage (%)
*Occupational sector*		
Construction sector	23	15.3
Textile industry	21	14
Healthcare sector	19	12.7
Automobile service	14	9.3
Administration	14	9.3
Food industry	8	5.3
Wood industry	7	4.7
Education	7	4.7
Hygiene	7	4.7
Electricity repair	5	3.3
Chemical industry	5	3.3
Metallurgy	4	2.7
Trade	4	2.7
Hair dressing	3	2
Agriculture	3	2
Tourism	3	2
Painting	2	1.3
Public transportation	1	0.7

*Occupational consequences*		
No change	117	78
Transfer	15	10
Decline of incomes	8	5.3
Work loss	7	4.7
Change of company	2	1.3
Reclassification	1	0.7

**Table 2:** Medical characteristics of patients.

Variables	Number of cases	Percentage (%)
*Clinical presentation of ACD*		
*Acute lesions*	31	20.6
erythemato-vesicular	29	19.3
bullous	2	1.3
*Subacute eczema*	27	18
erythemato	13	8.6
scaling	14	9.3
*Chronic eczema*	11	7.3
keratotic	3	2
lichenified	2	1.3
cracks	6	4
*Associated forms*	81	54

*Symptoms *		
None	39	26
Itch	46	30.7
Burning	1	0.7
Pain	2	1.3
Others	2	1.3
Associated symptoms	62	41.3

*Extent of injuries*		
1%	37	24.7
2-9%	21	14
10-18%	41	27.3
19-29%	20	13.3
≥ 30%	31	20.7

*Number of relapses per year *		
None	3	2
1-5 relapses	75	50
6-10 relapses	22	14.7
11-20 relapses	7	4.7
Countless	43	28.7

*Prescription of treatment*		
Yes	140	92.7
No	10	7.3

**Table 3:** Association between poor QOL and variables studied after multiple linear regression.

	Initial Model				Final Model			
Variables	B	*p*	95% confidence interval		B	*p*	95% confidence interval	
			Inferior limit	Superior limit			Inferior limit	Superior limit

Female	0.16	0.04	0.005	1.81				
Age range	0.05	0.43	-0.22	0.51				
School level	<10^−3^	0.99	-0.42	0.42				
Lifestyle	0.04	0.52	-0.65	1.28				
Atopy	0.10	0.12	-0.23	1.83	0.13	*0.03*	0.09	2.008
Localization	-0.01	0.78	-0.50	0.38				
Clinical forms	-0.05	0.44	-0.27	0.12				
Number of relapses >10	0.10	0.13	-0.08	0.50	0.14	*0.02*	0.04	0.56
Work loss	0.08	0.24	-0.75	2.91				
Presenteeism	0.37	<10^−3^	-0.27	0.12	0.36	*<10* ^*-3*^	0.01	0.04
Absenteeism	-0,25	0,39	-0,09	0,03				
Daily activity impairment	0.12	0.16	-0.44	2.60	0.18	*0.03*	0.15	3.04
Overall work productivity loss	0.44	0.12	-0.01	0.09	0.18	*0.01*	0.004	0.03

*P*: degree of significance.

B: regression coefficient.

**Table 4:** Association between absenteeism and variables studied after multiple linear regression.

	Initial Model				Final Model			
Variables	B	*p*	95% confidence interval		B	*p*	95% confidence interval	
			Inferior limit	Superior limit			Inferior limit	Superior limit

Age range	0.006	0.75	-0.71	0.98				
Scholar level	0.01	0.61	-0.81	1.38				
Activities sectors	<10^−3^	0.98	-0.17	0.17				
Family history of eczema	-0.001	0.45	-6.12	1.52				
Clinical forms	-0.001	0.95	-0.5	0.47				
Localization	-0.005	0.78	-1.26	0.95				
Extent	0.05	0.008	0.24	1.54	0.04	*0.03*	0.06	1.28
Consequences	0.03	0.062	-0.04	1.6				
Treatment	-0.02	0.12	-6.12	0.75				
DLQI	-0.03	0.14	-0.7	0.10				
Presenteeism	0.06	0.01	0.01	0.09	0.07	*<10* ^*-3*^	0.02	0.08
Overall work productivity loss	0.92	<10^−3^	0.73	0.8	0.92	*<10* ^*-3*^	0.73	0.80
Daily activity impairment	0.02	0.28	-1.68	5.76				

*P*: degree of significance.

B: regression coefficient.

**Table 5:** Association between presenteeism and variables studied after multiple linear regression.

	Initial Model				Final Model			
Variables	B	*p*	95% confidence interval		B	*p*	95% confidence interval	
			Inferior limit	Superior limit			Inferior limit	Superior limit

Female	-0.026	0.71	-10.5	7.19				
Medical history	0.07	0.22	-1.47	6.24				
Lifestyle	-0.04	0.47	-4.60	2.15				
Activities sectors	-0.04	0.44	-0.91	0.41				
Localization	0.02	0.66	-3.42	5.39				
Clinical forms	0.03	0.53	-1.37	2.63				
Occupational consequences	0.07	0.20	-1.17	5.41				
Number of relapses > 10	-0.05	0.41	-4.16	1.71				
Treatment	-0.10	0.07	-26.6	1.31	-0.10	0.05	-26.25	0.08
Time between patch tests and diagnosis	-0.11	0.05	-6.26	0.08	-0.12	*0.02*	-6.13	-0.51
DLQI	0.24	0.001	1.19	4.40	0.25	*<10* ^*-3*^	1.39	4.37
Overall work productivity loss	0.20	0.003	0.07	0.35	0.20	*0.001*	0.08	0.34
Daily activity impairment	0.46	<10^−3^	32.8	58.99	0.45	*<10* ^*-3*^	32.48	57.41

*P*: degree of significance.

B: regression coefficient.

## Data Availability

The data used to support the findings of this study are available from the corresponding author upon request.
